# Impact of a mobile phone-based interactive voice response software on tuberculosis treatment outcomes in Uganda (CFL-TB): a protocol for a randomized controlled trial

**DOI:** 10.1186/s13063-021-05352-z

**Published:** 2021-06-13

**Authors:** Dathan Mirembe Byonanebye, Hope Mackline, Christine Sekaggya-Wiltshire, Agnes N. Kiragga, Mohammed Lamorde, Elizabeth Oseku, Rachel King, Rosalind Parkes-Ratanshi

**Affiliations:** 1 Makerere University School of Public Health, P.O. Box 7072, Kampala, Uganda; 2 Infectious Diseases Institute, Makerere University, P.O. Box 22418, Kampala, Uganda

**Keywords:** Interactive voice response, mHealth, tuberculosis, Resource-limited settings, Low - and middle-income countries, Africa

## Abstract

**Background:**

Throughout the last decade, tuberculosis (TB) treatment success has not surpassed 90%, the global target. The impact of mobile health interventions (MHIs) on TB treatment outcomes is unknown, especially in low- and middle-income countries (LMICs). MHIs, including interactive voice response technology (IVRT), may enhance adherence and retention in the care of patients with tuberculosis and improve TB treatment outcomes. This study seeks to determine the impact of IVRT-based MHI on TB treatment success (treatment completion and cure rates) in patients with TB receiving care at five public health facilities in Uganda.

**Methods:**

We used a theory-based and human-centered design (HCD) to adapt an already piloted software to design “Call for life-TB” (CFL-TB), an MHI that utilizes IVRT to deliver adherence and appointment reminders and allows remote symptom reporting. This open-label, multicenter, randomized controlled trial (RCT), with nested qualitative and economic evaluation studies, will determine the impact of CFL-TB on TB treatment success in patients with drug-susceptible TB in Uganda. Participants (*n* = 274) at the five study sites will be randomized (1:1 ratio) to either control (standard of care) or intervention (adherence and appointment reminders, and health tips) arms. Multivariable regression models will be used to compare treatment success, adherence to treatment and clinic appointments, and treatment completion at 6 months post-enrolment. Additionally, we will determine the cost-effectiveness, acceptability, and perceptions of stakeholders. The study received national ethical approval and was conducted in accordance with the international ethical guidelines.

**Discussion:**

This randomized controlled trial aims to evaluate interactive voice response technology in the context of resource-limited settings with a high burden of TB and high illiteracy rates. The software to be evaluated was developed using HCD and the intervention was based on the IMB model. The software is tailored to the local context and is interoperable with the MHI ecosystem. The HCD approach ensures higher usability of the MHI by integrating human factors in the prototype development. This research will contribute towards the understanding of the implementation and impact of the MHI on TB treatment outcomes and the health system, especially in LMICs.

**Trial registration:**

ClinicalTrials.govNCT04709159. Registered on January 14, 2021.

## Introduction

The burden and impact of TB on low- and middle-income countries (LMICs) is unprecedented. TB is the leading infectious cause of death worldwide [1]. The disease is one of the leading causes of hospitalization [2] and in-hospital mortality in sub-Saharan Africa [2, 3]. LMICs contribute more than 80% to the global incidence of TB [1]. The high burden in LMICs is mainly attributable to HIV [4], poverty [5], and weak health systems [6]. Despite the high burden, health systems in LMICs are unprepared to contain the epidemic. Adherence and retention in TB programs are low [7, 8], which hampers TB control efforts. Modeling studies suggest that strengthening the TB care cascade could reduce TB incidence by more than 30%. However, there are limited data on the role and impact of mobile health interventions (MHI) in strengthening TB care systems [9].

Mobile phone use and access to the Internet are associated with a reduction in TB incidence and mortality [10]. The impact of mobile health technologies on the enhancement of infectious disease services has been demonstrated globally [11, 12]. The WHO’s “End-TB strategy” emphasizes the use of MHIs to accelerate the implementation and monitoring of TB services [13] and has recommended the application of MHIs to improve adherence to treatment [14]. The rapid growth in the mobile phone industry has transformed health systems across many LMICs [15–17]. Therefore, the increasing mobile phone ownership in LMICs, including in HIV/TB clinics [18, 19], offers an opportunity to strengthen health systems and ensure the delivery of patient-centered care. This may improve patient retention in care and treatment adherence, ultimately improving TB treatment outcomes [20]. MHIs are acceptable to people with chronic infections in several LMICs [21–23], and the infrastructure for their use is improving in many LMICs, including Uganda [24].

**Fig. 1:**
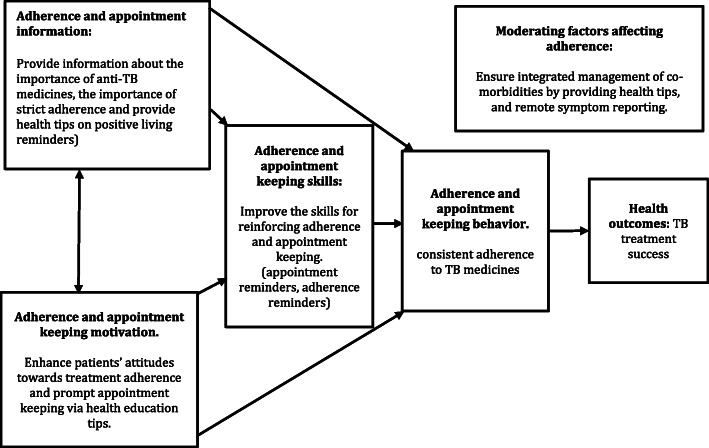
Behavioral change conceptual framework for the study, adapted from Informational Motivation- Behavioral skills model (Fisher, W. A., & Fisher, J. D. (2009))

The majority of phones used in LMIC are feature phones (non-smart), which are not compatible with the technologies that have been demonstrated to enhance TB care in developed countries [25]. In LMICs, the evidence for the impact of MHIs on TB treatment outcomes is limited to studies involving short message service (SMS) interventions. Although SMS interventions seem to be generally associated with higher treatment success [26], the results from LMICs have been contradictory. Some studies have reported a significant impact on treatment success [12] while others did not find better treatment outcomes with SMS interventions [27–29]*.* The implementation of SMS MHIs is low in LMICs because of high illiteracy and preference for voice-based communication [30–32]*.* Interactive voice response technology (IVRT) delivers voice calls that do not require reading skills. Therefore, IVRT may be acceptable and may have a greater impact. While there is increasing evidence for use of IVRT to optimize care in people receiving HIV care and other health services [33–35], no study has evaluated the impact of mobile-based IVRT on TB treatment outcomes in Africa. We used the information, motivation, and behavioral skills (IMB) model of behavioral change [36] to guide the design of an IVRT-based technology that enhances adherence to TB treatment. The IMB model was initially developed to modify HIV-risky behaviors and has now been adopted to guide behavioral change in developed countries [34]. The model suggests that the motivation of TB patients, increasing adherence skills, and providing information on TB treatment would improve adherence. The model was used to target increase adherence and retention in care since improving these aspects may increase treatment success (Fig. 1).

Owing to the novel approach of IVRT for TB, in this paper, we document our protocol to inform the body of work around the MHI for TB. Furthermore, given the complexity of MHI and its capacity at multiple behavioral levels, we will be undertaking an interdisciplinary study with qualitative, quantitative, and economic components.

### Research objectives

The primary objective of this study is to determine the impact of an IVRT software (CFLU-TB) on treatment success in patients receiving treatment at public facilities in Uganda. The secondary objectives were as follows:

1. To compare TB cure rates (six months) in patients with microbiologically diagnosed TB in the intervention and control arms.
2. To compare treatment completion (six months) in patients clinically diagnosed with TB in the intervention and control arms.
3. To determine the effect of enhanced TB treatment support with CFLU-TB on retention at 2 and 6 months in patients receiving TB treatment.
4. To assess the perception of patients’ care providers (treatment buddies) and other stakeholders about CFLU-TB.
5. To determine the cost-effectiveness of the intervention.
6. To compare adherence rates between patients in the intervention arm and control arms.
7. To determine the effect of CFLU-TB on adherence to clinic appointments.
8. To compare knowledge about TB/HIV in patients enrolled in the intervention and control arms.
9. To determine Drug-resistant TB rates in the intervention and control arms.
10. To determine the uptake of the CFLU-TB tool in patients and their care providers (treatment buddies).

### Study hypothesis

We hypothesized that IVRT will have a 10% higher impact on treatment success than SMS intervention; therefore, we predict that CFL-TB will lead to a 15%-point increase in treatment success rate in the intervention arm compared to the standard of care (control arm). This is based on a previous RCT that reported 5–9% better treatment success in patients who received SMS adherence reminders versus the standard of care [12, 27].

## Methodology

### Trial design

This study will be an open-label, multicenter RCT involving adult patients with drug-susceptible TB randomized (1:1 ratio) to either the intervention (adherence and appointment reminders, health messages, and 24-h health care worker call service) or control arm. The study will have nested qualitative and economic evaluation sub-studies and a process evaluation to determine the uptake and use of the intervention.

### Study settings

To ensure the generalizability of the results of this study, we planned to enroll patients from several facilities that are representative of typical TB facilities in Uganda. Most patients with tuberculosis [37, 38] are treated at low-level public facilities but a significant burden also exists in urban settings [37, 38]. Therefore, we selected both public and rural TB treatment facilities. Kisenyi Health Center, the first study site, is a large public health center situated within the oldest slum in Kampala which is home to over 23,000 people vulnerable to TB. As a result, the facility receives about 25 new TB patients per month. In addition, Kiryandongo is a rural agrarian district hospital that serves 266,000 people, including 50,000 refugees. The hospital registers an average of 9 new TB cases per month. In contrast, Kasangati Health Center IV is a busy peri-urban facility that serves the Kyadondo East in the Wakiso district and attends to approximately 24 new TB cases per month. To expedite participant accrual, two additional facilities were added to the study protocol version 2.0. Bweyogerere and Kawanda Health Centre IIIs are in the Wakiso district and serve a peri-urban population. They treat approximately 12 patients with TB each per month.

At all health facilities [39, 40], patients with at least one of the dangerous symptoms of TB are offered a sputum GeneXpert test while those unable to expectorate are offered alternative diagnostic tests (CXR, TB LAM, sonography, tissue biopsy, CSF analysis, etc.). Patients with sputum-positive TB are then tested for rifampicin resistance using the GeneXpert MTB Rif assay [40].

### Control arm and the standard of TB care in Uganda

The standard of care at all TB treatment centers follows the national TB treatment guidelines [40]. The standard treatment duration is 6 months, except for patients with TB meningitis and osteoarticular TB who require 12 months of treatment. The standard regimen and duration of treatment for patients with drug-susceptible TB are 2 months for isoniazid, ethambutol, rifampicin, and pyrazinamide, followed by four months of rifampicin and isoniazid [34]. Patients are reviewed every 2 weeks for the first month and monthly thereafter. All patients are encouraged to register a close household or community member to directly observe therapy, but this is not enforced. Patients receive health education during clinic visits with messaging on adherence and treatment completion. At treatment initiation, patients are routinely encouraged to adhere to treatment and appointments, but there are often no patient support systems provided to enhance this. Some patients may use other reminder systems of their choice [41] but this has not been described in patients with TB.

### Description of the proposed mobile health intervention

The technology to be evaluated in this study is Call for Life-TB (CFL-TB^TM)^, a software that is based on open-source mobile technology for community health (MoTeCH) [42]. MoTeCH was initially developed by the Grameen Foundation and the University of Southern Maine with the support of Janssen, the Pharmaceutical Companies of Johnson and Johnson. It was designed as a community MHI platform to improve treatment compliance in patients in LMICs [42, 43]. We then adapted and evaluated the technology to support people living with HIV in Uganda and named it Call for Life ^TM^ (CFL) [34]. CFL-TB uses interactive voice response (IVR) or short messaging service (SMS) to communicate with PLHIV via mobile phones [34]. This prototype, “Call for Life-Uganda”, was iteratively adapted to design CFL-TB, a technology tailored to address the needs of patients with TB. The needs of patients with TB were enumerated through discussions with health workers and patients at IDI HIV/TB clinics. The software was sequentially upgraded, and the subsequent versions were reviewed by end-users at each stage. End-users (study staff and patients with TB) reviewed the software for suitability and suggested changes. Overall, the changes were aimed at optimizing the usability and security of the software. Participants in the intervention arm will receive standard of care plus CFL-TB enhanced TB care, and those in the control arm will receive standard of care.

### The intervention (CFL-TB)

CFL-TB delivers once-daily adherence reminders, pre-appointment reminders, and targeted educative behavior change messages and allows patients to report symptoms to health care workers (HCWs) remotely. As advised by Iribarren et al., participants will decide their preferred language and the timing and frequency of reminders and will ensure that the content is culturally sensitive [44]. The software also delivers calls to registered care providers (buddies) of patients to facilitate daily observed therapy (DOTS), in line with the World Health Organization and Ministry of Health guidelines [14, 40]*.* In Uganda, daily reminders are generally preferred by patients over weekly reminders [8]. To increase adherence to the intervention, if the first call is not answered, the system will make two additional calls, 15 min apart. The study staff will also interview participants in the intervention arm to determine whether they are experiencing any problems. Health care workers enroll patients in the system, monitor the delivery message delivery of messages, and provide ongoing technical support, such as setting personal identification numbers (PINs) and swapping the registered telephone numbers. In contrast, participants are responsible for making and receiving calls. We will synchronize appointment reminders with scheduled appointments, and the software will initiate a call before and on the day of the clinic review. Participants select their preferred time to receive the reminder calls and set their PIN. The system offers short educational messages to increase knowledge about TB/HIV and general health [40]. The CFL-TB software is interoperable with other health information systems used in Uganda, including DHIS2, a web-based database for health reporting.

### Study population and eligibility criteria

**Fig. 2:**
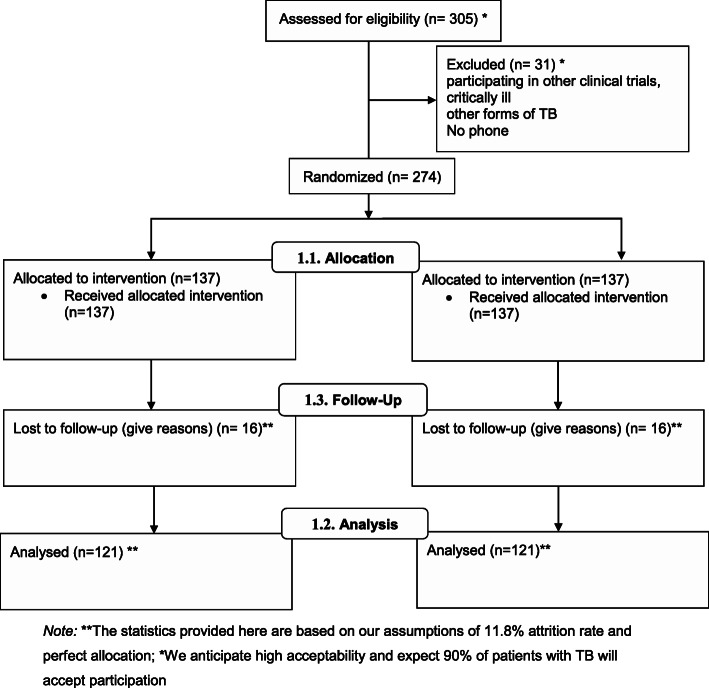
CONSORT Flow Diagram

This study will enroll adults (≥ 18 years) who were newly diagnosed with drug-susceptible TB (Fig. 2). The detailed eligibility criteria include the following:

#### Inclusion criteria

1. Evidence of TB diagnosis either confirmed bacteriologically by Xpert MTB/RIF Version G4 assay (Cepheid, Sunnyvale, CA, USA) or clinically diagnosed.
2. Evidence of a personally signed and dated informed consent document that indicates that the participant (or a legal representative) has been informed of all pertinent aspects of the study.
3. Willingness to comply with scheduled visits, treatment plans, laboratory tests, and other study procedures.
4. Aged ≥ 18 years
5. Mobile phone ownership. Subject to funding, study participants without mobile phones may be provided with mobile phones.
6. Patients who can understand Luganda, Runyankore, Swahili, or English. These languages are the languages spoken by most patients at the study health facilities. If we add Lira RRH as a study site, we will add Luo, the predominant language spoken in the Lango region. Other languages may be added to the service if the anticipated demand surpasses 30 patients.

#### Exclusion criteria

1. Inability to use a basic feature phone or presence of a clinical condition that interferes with appropriate use of the mobile phone for voice calls (e.g., deafness, severe cognitive impairment)
2. Ongoing participation in another interventional study that the investigator believes will interfere with study procedures or assessment of the outcomes of this study.
3. Patients who are critically ill.
4. Patients with drug-resistant TB (Rifampicin resistant, Multi-drug resistant, and extensive drug-resistant TB).
5. Patients with TB meningitis or osteoarticular TB.
6. Any other clinical condition that, in the opinion of the site investigator, would make the participant unsuitable for the study or unable to comply with dosing requirements.

### Recruitment of study participants and strategies for achieving adequate participant enrolment

This study will recruit from TB clinics within the study clinics. The study staff will identify participants newly diagnosed with TB. To expedite participant accrual, two additional facilities have been added to the study protocol version 2.0. Bweyogerere and Kawanda Health Centre IIIs are in the Wakiso district and serve a peri-urban population. They receive an average of 13 and 10 patients with TB per month (approximately 156 and 120 patients per year, respectively). Additionally, study initiation meetings have been conducted with health facility staff and patients at all study sites study to increase awareness about the study. We hope this enhances the referral of participants to the study.

### Randomization, blinding, and allocation concealment

At baseline, participants will be randomly assigned (1:1 ratio) to either the intervention or control arms (Fig. 2). The study statistician generated study allocation slips for participants for each study site using and these are then enclosed in sealed opaque envelopes. The number of cards allocated per study site will be equal to the enrolment target assigned to the study site. The allocation slips will indicate either the intervention or the control arm. The study arm will be allocated by drawing one envelope by a participant from a closed envelope box. Patients will randomly pick and open an envelope and they will be allocated a study arm that is indicated on the enclosed card. Since the opaque envelopes will be used and drawn from a closed opaque box, the allocation sequence is concealed from the recruiting study staff. We will maintain screening and enrolment logs at each study site to ensure that there is no bias in randomization and enrolment. Due to the study design involving voice reminders to participants, it is not possible to blind participants. However, the analyzing statistician will be blinded to the allocated arms.

### Participant follow-up, procedures, and timelines

Participant follow-up before six months, the total study duration is 24 months, and participants will have evaluations at baseline and months 2, 5, and 6, consistent with the Ministry of Health visit scheduling for patients with TB (Table 1). At each visit, the participants will be given their future appointment dates.

**Table 1:** Study timelines and procedures

Study procedures	Study procedure completed							
Month	0	0	2	2	5	5	6	6
Study arm	I	C	I	C	I	C	I	C
Screening	**√**	**√**						
Informed consent	**√**	**√**						
Physical evaluations and interviews	**√**	**√**	**√**	**√**	**√**	**√**	**√**	**√**
Demographic and socio-economic questionnaire	**√**	**√**	**√**	**√**	**√**	**√**	**√**	**√**
Sputum GeneXpert^*^	**√**	**√**			**√**	**√**	**√**	**√**
Drug culture and susceptibility tests^β^	**√**^β^	**√**^β^	**√**^β^	**√**^β^	**√**	**√**	**√**^β^	**√**^β^
Cost-effectiveness questionnaire	**√**	**√**	**√**	**√**	**√**	**√**	**√**	**√**
Qualitative assessment	**√**	**√**	**√**	**√**			**√**	**√**

Note: I-Intervention arm, C-control arm

^*^Additional tests to ascertain sputum conversion may be added at the discretion of the investigator.

^β^Drug susceptibility testing will be done in-line with national TB guidelines. Sputum cultures may be done at 2 months to ascertain sputum conversion and sputum for genotyping TB stored.

**√** indicates procedure completed at the indicated timeline

### Participant withdrawal and post-trial care

Participants can be withdrawn from the RCT at their request, following death or disengagement from care. Participants may also be withdrawn at the discretion of the investigator for safety concerns. If the subject withdraws from the RCT, the study staff will not perform further evaluations, and data collection will cease. The RCT will retain data collected before withdrawal unless the participant requests otherwise. Study staff will track participants who miss visits. If a participant does not attend for two consecutive months, they will be considered lost to follow-up (attrition). All withdrawn participants will be linked to the standard of care. Participants with cured TB will be exited from care while those with no evidence of cure will be linked to tertiary TB services at their treatment facilities. At the end of the RCT, participants will have their treatment details updated in the TB register and are discharged from care.

### Data collection and management

We will comply with the Ugandan laws on data privacy and management [45]. Data will be entered using REDcap [46]. The system automatically flags missing and out-of-range values, which will be attended to by the RCT staff.

### Data collection

At baseline, the staff will obtain sociodemographic data (age, sex, marital status, level of education, employment status, income, transport costs, distance from the facility) and data on TB infection (method of diagnosis, site of infection, and rifampicin resistance at baseline). Prospectively, data will be obtained on adherence, symptoms, sputum tests, missed appointments, and clinic visits (Table 1). Participants will also be interviewed on phone call costs, hospitalization, and over-the-counter treatments. At baseline, we will interview patients (qualitative data) on their anticipated challenges in the use of IVRT and anticipated benefits and challenges. At months 2 and 6, qualitative data will be collected on acceptability and experience with the digital intervention. We will determine the sputum conversion rate at 2 months and cure at 6 months. Chest radiographs will be used to determine the cure for patients with clinical TB. TB cards and treatment registers will be reviewed for death, adherence, and date of patient visits. We will use micro-costing to obtain data on the direct and indirect costs.

### Trial endpoints

The primary endpoint is treatment success (at 6 months): treatment success is a composite outcome, comprising treatment completion and cure in patients with clinically and bacteriologically diagnosed TB, respectively. It is a robust and agreed indicator of treatment success in TB programs. In patients with bacteriologically diagnosed TB, cure is defined as having a negative sputum test in the last month of treatment and on at least one previous occasion [47]. For clinically diagnosed patients, completion entails 6 months of therapy. The denominator for each arm will be the number of patients enrolled. At the same time, the numerator is the sum of bacteriologically and clinically diagnosed patients with cure or treatment completion, respectively.

This RCT also has several secondary endpoints. First, we will determine the TB cure rates in patients with microbiologically diagnosed TB. Second, we will determine treatment completion for patients clinically diagnosed with TB in the two arms: Third, retention rates will be determined and compared between the study arms. Patients will be deemed to be actively engaged in care if they attend a clinic visit within one month of a scheduled appointment. Additionally, we will compare appointments. We will categorize visits as “early,” if they present before an appointment, “prompt” if they show up within three days after the appointment date, “late” if they present after three days but within 30 days of a scheduled appointment and missed if patients do not show up at all or present after 30 days. Fourth, we will determine the incremental costs per additional DALY averted per percentage point increase in treatment success in the intervention versus the control arm. In addition, we will compare adherence rates at months 2 and 6. Depending on the availability of drug susceptibility tests, we will compare the rates of MDR-TB. To understand the impact of the intervention on the treatment outcomes and the potential for adoption and scaling of the intervention, we will explore the perceptions and experiences of patients and care providers towards the MHI. Finally, we will determine the uptake and adherence to the intervention. We will consider the successful receipt of a reminder as a proxy for the utilization of the technology. Therefore, we will determine the proportion of successful calls in the intervention and control arms.

### Sample size determination

Based on results from a randomized controlled trial in Kenya that demonstrated a 9% increase in TB treatment success following the use of SMS [12], we hypothesize that the interactive voice response platform will lead to at least a 6% higher impact as compared to SMS reminder treatment success (i.e., a 15%-point difference compared to the control arm). A retrospective cross-sectional study of routine data on HIV/TB co-infected patients from the Uganda National Tuberculosis and Leprosy Program reported lost to follow-up rates of 3.5% and 16.6% in urban and rural settings, respectively [7]. The rate of lost-to-follow at the three TB clinics was approximately 11.8%. Therefore, assuming an 11.8% lost-to-follow-up rate, this study would require a minimum of 274 (137 patients per arm) patients to demonstrate a 15% increment in treatment success with a power of 0.8 and precision of 0.05.

### Analysis of endpoints

The planned analyses are summarized in Table 2. We will use a two-tailed *p* value of 0.05 and report the 95% confidence intervals for all endpoints. In all analyses, we will control for confounders of adherence and retention [7, 48, 49]. In both primary and secondary outcomes, we will undertake per-protocol and intention-to-treat analyses.

**Table 2:** Analysis of quantitative outcomes

Outcomes	Measure of outcomes (intervention versus control arm)	Statistical analysis
Treatment success	Proportion of patients with treatment success at six months. Odds ratio of treatment success	Logistic regression, odds ratios
Cure rates	Proportion of patients cured of TB; odds ratio of TB cure	Logistic regression, odds ratios
Treatment completion	Treatment completion rate; odds ratio of treatment completion	Logistic regression, odds ratios
Retention in care	Hazards of attrition in the intervention versus control; Cumulative attrition at six months	Cox’s proportional hazards model, log-rank test
Adherence to TB medicines	Mean adherence rates (proportion of TB medicines)	Mann-Whitney U test and Poisson regression
Appointment keeping and punctuality	Proportion of patients who keep an appointment; Proportion of patients with early, prompt, and late visits	Ordered logistic regression
Drug-resistant TB rates	Proportion of patients with rifampicin-resistant TB	Logistic regression, odds ratios
Call success rates (2,4, and 6 months)	Proportion of successful calls	*t* test

### Analysis of the primary endpoint

We will use logistic regression to determine the crude and adjusted odds ratios of treatment success in the intervention arm versus the control arm. The multivariable model will adjust just for potential confounders, including age, sex, employment status, bacterial load at baseline, TB category, and HIV status [7, 48, 49]. The stepwise backward elimination method will be used to fit the final model. The model with the lowest Akaike information criterion (AIC) statistic will be considered the best fitting. Covariates with a global/trend *p* value > 0.2 at the bivariate analysis, will be adjusted for in the final multivariate model. We will explore factor interactions, including the interaction between age adherence and sputum smear results at baseline. Where interaction is confirmed, we report both the main and interaction effects.

### Analysis of quantitative endpoints

Logistic regression will be used to determine the adjusted odds ratio for TB cure and treatment completion in the intervention arm versus control arms. We will also determine the predictors of TB cure overall and in both arms. The final model will adjust for age, sex, bacterial load, HIV status, TB site, and symptoms at baseline. We will also determine and compare attrition in the two study arms using the Cox proportional hazards model. We will compute the crude and adjusted hazard rates of attrition in the intervention and control arms. The log-rank test will be used to test for differences in attrition between the study arms. Tests for the Cox proportional hazard assumption will be done graphically using Schoenfeld residuals. The model will adjust for age, sex, Karnofsky score at baseline, HIV status, transport costs, distance from the health facility, TB site, and socioeconomic covariates.

We will compare the mean adherence rates (proportion of TB medicines taken as evidenced by TB card) at 6 months in the intervention and control arms. We will treat adherence as a continuous covariate and compare the median adherence between the intervention and control arms. To compare appointment keeping, we will use ordinal logistic regression to determine the adjusted odds ratios of missed, late, and prompt visits (early used as a reference group). In the sensitivity analysis, Poisson regression will be used to compare the number of missed visits and treatment doses in the intervention and control arms. The models will adjust for age, sex, employment, distance from the health facility, and HIV status. We will use chi-square to compare the proportions of patients with MDR-TB between the two study arms. Additionally, logistic regression will be used to determine the crude and adjusted odds ratios of MDR-TB in the intervention and control arms. Finally, as a proxy for the uptake of intervention and fidelity, we will determine the proportions of successful and dropped and received calls at three different (2, 4, and 6 months) to determine patient exposure and acceptance of the intervention through the study treatment period.

### Cost-effective analysis

For the determination of cost-effectiveness, we will follow the WHO’s guidance on the economic evaluation of TB interventions [50]. To this end, we will determine the incremental costs per additional DALY averted per percentage increase in treatment success. All costs and DALYs derived from the models will be discounted at 3%, and a 5-year horizon will be used. The price year used will be the first year of research implementation, and a discount rate of 3% shall apply. We hope that this intervention will be scaled up if it shows clinical effectiveness. Therefore, we model the overall programmatic cost of implementing the intervention at scale.

We will use the societal perspective to determine the cost-effectiveness of the intervention, assuming government standard rates and costs. From the financial records of the project, we will abstract disaggregated data on the costs (prevailing dollar rate in the year of implementation) of direct financial costs (micro-costing). To determine the direct costs for scale-up, we will exclude costs related to conducting research that would otherwise not be met if the intervention is implemented according to government standard cost.

We then model the cost-effectiveness of the intervention by assuming 72% and 100% phone coverage in patients with TB. We will construct a decision-analysis model using TreeAge® software (version 3.5, MA, USA) to compare the cost-effectiveness of digital intervention versus standard of care. The probabilities of cure and treatment completion to be used in the software will be obtained from the study (for intervention) and national TB reports (for control arms). We will use the national TB prevalence survey [38] to estimate the number of patients with TB nationally. The years of life lost (YLLs) averted will be calculated by subtracting the median age at death from TB from the estimated average life expectancy at birth for Uganda. Regarding the determination of YLL for HIV co-infected patients, a Markov model for ART-experienced PLHIV will be fitted. The global burden of disease estimates will be used to determine the disability-adjusted life years (DALYs) weights for those patients with TB and life expectancy at birth [51].

### Qualitative analysis

We will explore the experiences of patients and stakeholders while using the CFL-TB tool through focus group discussions and key informant interviews. We will use purposive sampling and continue data collection until saturation is achieved [52]. Participants will be stratified by gender, age, and use of the tool. We will use the thematic analysis approach to determine what participants and other stakeholders experience and perceive. The conventional content analysis approach will be used; the coding categories will be derived directly from the text data [53]. Preliminary data analysis will occur concurrently with the data collection. The process of proofreading transcripts and notes will help draw attention to emerging and a priori themes. Themes then form a framework for a codebook. During the coding process, the codebook will be adapted and quotations that are illustrative of participants’ perspectives and experiences regarding the issues being studied will be identified and used in the presentation of the findings. Using the directed qualitative analysis approach [53], we will explore the relationships between the intervention and treatment outcomes using the IMB model [36] as a guide theory.

### Sensitivity analysis

To determine the impact of nonadherence to the study intervention, we will stratify the analysis by utilization of the CFL-TB tool. For both primary and secondary quantitative endpoints, we will conduct an overall and stratified analysis by HIV status, TB type (pulmonary versus other forms of TB), and gender.

### Interim analysis

There will be no preliminary analysis, as this study does not pose a significant risk to participants.

### Ethical considerations

The study protocol was approved by the Makerere University School of Medicine Research Ethics Committee (SOMREC) (Approval #:2019-149) and the Uganda National Council of Science and Technology (UNCST) ((Approval #: HS524ES). Any protocol amendments will be reviewed and approved by SOMREC and UNCST. Therefore, we will follow the UNCST guidelines [45, 54], the Declaration of Helsinki [55], and good clinical practice [56] during the study implementation. Before participation, the study staff will screen and obtain written informed consent from all prospective participants and their care providers. There will be no additional requirement for consent before obtaining biological specimens, including blood samples and sputum, as these are part of the standard of care.

### Protocol amendments

The initial protocol (version 1.3, approval date November 07, 2019) (Supplementary File 1) was amended, and protocol version 2.0 was approved by SOMREC and UNCST on March 22, 2021. The major changes included a provision for phone-based reviews and an additional two study sites. Supplementary File 2 provides details of the changes made in protocol version 2.0. The latest approved protocol version 2.0 is provided as Supplementary File 3.

### Confidentiality of participant data

To ensure the safety of the participant data, data are regularly entered into a database hosted on the IDI server. The data on the CFL-TB backend were encrypted. Access to the system database is password-protected and inactivity for more than 30 min on the CFL-TB web interface logs the web user out. The system generates a log of admin logins and the changes to the web interface. Patients calling on CFL-TB are authenticated by their registered mobile number and must key-in their PINs before listening in to the calls. The software does not reveal its own identity to the person called until the person has been authenticated as a registered patient (only CFL-TB signature music is played). The Analysis datasets will be anonymized before data analysis by assigning participants with pseudo identification numbers (IDs) delinked from the study IDs. Phone call records will not be disclosed but may be used for matching data.

### Adverse events reporting

At each visit, participants will be interviewed on any ongoing symptoms and hospitalizations since their prior visit. The CFL-TB platform will maintain a patient toll-free number, and patients will be asked to initiate calls when they want to communicate with HCWs. All adverse events (AEs) will be graded using the Division of AIDS (DAIDS) table for adverse events [57]. Serious adverse events (SAEs) will be reported to IRBs. The investigator will assess the relatedness and causality of AEs to mobile phone use.

### Study oversight and monitoring

The design, implementation, and evaluation of this study will be monitored by an independent scientific advisory board (SAB). The SAB comprises eminent community members, policymakers, scholars, and scientists. There will be no data safety monitoring board, as the study does not pose a significant risk to participants. Any suggested protocol amendments will be reviewed by the SAB before submission to IRBs for approval. The IDI research department will monitor the study progress annually.

### Dissemination plan for research findings

Dissemination of the results of this study will follow the institutional dissemination policy (IDI) and national regulations on data privacy [45]. We aim to publish the results and present data at national, regional, and international conferences. The study has been registered with clinicaltrials.gov (NCT04709159). Since we aim to influence the TB policy in Uganda and other LMICs, we will present the results at local meetings with policymakers. Policy briefs will be presented to the National TB and Leprosy Program of the Ministry of Health. We will also conduct community dissemination meetings and communicate the results of this study to the participants and care providers.

## Discussion

This study is a multicenter, open-label, randomized controlled trial to determine the impact of IVRT-based MHI on TB treatment outcomes. The study will compare treatment success at six months in participants with confirmed drug-susceptible TB randomized to the intervention and control arms. The secondary outcomes include TB cure rates in participants with pulmonary TB, appointment keeping, adherence to treatment, and attrition from care. Participants in the intervention arm will receive adherence and appointment reminders and health tips and will also have options for reporting symptoms remotely. We will also use mixed methods to determine the acceptability of the intervention, its cost-effectiveness, and its impact on TB treatment outcomes.

This study is not without justification. In 2018, there were 10 million cases of TB, with 1.45 million deaths, 250,000 of whom were patients living with HIV/AIDS (PLHIV) [1]. The disease has also harmed other health sectors [58]. However, whether MHIs can significantly contribute to a sustainable TB response is unknown. The growth of the mobile phone industry is highest in countries with weak health systems and a high burden of TB [59]. Mobile phone ownership is also high in patients with TB [19], especially among men, the population most affected by TB [1, 38]. Therefore, the delivery of the intervention at the population level is feasible.

In most LMICs, the *sine qua non* for the success of MHI is the implementation fidelity and tailoring the technology to the local context. Previous studies have demonstrated that technical difficulties are a barrier to the use and scale-up of MHIs. These include power outages and sparse network signals, especially in rural communities [33, 60]. Additionally, only 55% of Uganda is geographically covered by the Internet [24]. Thus, MHIs that have previously been demonstrated to be efficacious, including video technology [25], are not implementable in many LMICs.

To enhance the scale-up of MHIs, the proposed interventions must be interoperable with existing MHIs and technologies. To this end, the Ugandan government requires MHIs to be sustainable, compliant with data privacy, and interoperability with existing MHIs [61]. The IVRT under investigation meets this requirement as it is interoperable with DHIS2, the only national database for health reporting in Uganda. Additionally*,* confidentiality concerns are common among all MHIs. A study involving the use of IVRT to support the adoption of TB preventive therapy in Ethiopia has reported confidentiality, stigma, and trust in HCWs as critical determinants of the acceptability of MHIs [33]. However, concerns about privacy and refusal to participate in MHIs are generally low in Uganda [28, 62]. The Sharing of mobile phones outside households is also rare [63]. However, MHIs have an inherent risk of data privacy breaches that must be mitigated. Therefore, data will be double-encrypted, system upgrades will be version controlled, and participants will use PIN before receiving reminder calls.

Although several frameworks for mobile health application development exist, HCD is increasingly preferred in resource-limited settings [64, 65]. The HCD approach ensures higher usability of interactive systems by integrating human factors such as human knowledge and techniques in the prototype development [66]. This approach has been used to develop other TB health information systems in sub-Saharan Africa [65]. As emphasized by the principles of HCD [64], software development involved a multidisciplinary team and broader stakeholder engagement. Such an approach ensures the uptake and scaling of MHI in LMICs [32].

We envisage the challenges in the implementation of this study. In a pilot project that evaluated the use of IVRT to enhance access to child health care services in Ghana, the critical barriers to the adoption of the IVRT were lack of human interaction, lack of refresher training, and the lack of social integration of the system [35]. Therefore, we will conduct regular refresher training for staff. Similarly, the study staff will interview participants at every study visit and ensure they have no challenges in using the tool. The software is compatible with feature phones which have the greatest penetration in Uganda [67]. On the other hand, the significant reasons for the success of the pilot project were the ability of the technology to support local languages, quality of the service, health education, and empowerment of end-users. Daftary et al. also reported individualizing MHIs as an enabler of acceptability [33]. To this end, we will individualize appointment and adherence reminders by allowing patients to select the preferred reminder frequency and their timing. However, contextualizing intervention is very difficult to achieve in Uganda because of cultural diversity [68] and limited technology options. We used several major local languages spoken in Uganda. Our IVRT allows for two-way communication and therefore allows human interaction. In the implementation phase of the project, we will offer refresher training to patients and staff on how to use the technology.

## Trial status

The initially approved protocol was version 1.3 (version date: October 07, 2019) but this was superseded by protocol version 2.0 (version date: March 22, 2021). The amendments to the protocol in protocol 2.0 added two additional study sites and provided for phone-based phone review during the COVID epidemic. The latter is to ensure there is minimal congestion in health facilities. Consistent with Uganda Ministry of Health recommendations for COVID. The CFL-TB trial has received approval from SOMREC and UNCST. Enrolment for this study commenced in October 2019 and has so far enrolled 123 of the required 274 participants as of May 15, 2021. Study enrolment is expected to end in December 2021 and study results will be available by the end of 2022.

## Conclusion

The CFL-U TB study seeks to determine the impact of IVRT-based MHI on TB treatment outcomes in a high-burden country. The software aims at addressing the major operational barriers to the optimization of TB care by leveraging technology with a broader reach regardless of literacy levels. The enrolment of study participants has commenced, and preliminary study results will be expected by the end of 2022. Like other MHIs, we anticipate several challenges, but these are not insurmountable. The changing mobile technology ecosystem and the increasing mobile phone penetration are possible feasible solutions to health system challenges in most low- and middle-income countries. However, the impact of such technologies remains to be determined. While there are design and implementation limitations, this study addresses a significant public health problem using a novel hitherto used MHI.

## Data Availability

The study investigators will keep custody of the data on the local servers based at IDI. Upon study conclusion, the findings and data from this study are available from the authors, but restrictions apply to the availability of these data. The Uganda Data Protection and Privacy Act, 2019, expressly prohibits sharing and hosting research and clinical data in the cloud. Therefore, the data may not be publicly available. Data are however available from the authors upon reasonable request and with permission from the government data protection office. Nevertheless, we have provided copies of important study documents, including the study protocols.

## Abbreviations

AE: Adverse events
DOTs: Directly observed treatment
MHIs: Mobile health interventions
DR-TB: Drug-resistant TB
ECG: Electrocardiogram
FGDs: Focus group discussions
HIV: Human immunodeficiency virus
IDI: Infectious Diseases Institute
IRB: Institutional review board
IVR: Interactive voice response
IVRT: Interactive voice response technology
LMICs: Low- and middle-income countries
MoH: Ministry of health
MDR: Multi-drug resistant TB
MHIs: Mobile health interventions
MOTECH: Mobile Technology for Community Health
PLHIV: People living with HIV/AIDS
SAE: Serious adverse events
SMS: Short messaging service
SOMREC: Makerere University School of Medicine Research Ethics Committee
TB: Tuberculosis
UNCST: Uganda National Council for Science and Technology
WHO: World Health Organization
